# Identification of endoplasmic reticulum stress-related biomarkers in asthma and asthma exacerbation

**DOI:** 10.3389/fmed.2026.1840272

**Published:** 2026-07-27

**Authors:** Lingling Xuan, Yali Lv

**Affiliations:** Ethics Office, Beijing Chao-Yang Hospital, Capital Medical University, Beijing, China

**Keywords:** asthma, endoplasmic reticulum stress, *ERN1*, exacerbation, *TMEM129*

## Abstract

**Background:**

Endoplasmic reticulum (ER) stress plays a crucial role in airway inflammation and other asthma-related processes. This study aims to identify potential ER stress-related biomarkers in asthma.

**Methods:**

Sputum transcriptome data (GSE76262) from 118 moderate-to-severe asthma patients and 21 healthy controls were analyzed to identify ER stress-related differentially expressed genes (DEGs). Functional enrichment, weighted gene co-expression network analysis (WGCNA), and protein-protein interaction (PPI) network construction were performed. Machine learning algorithms (LASSO logistic regression and random forest) were applied to screen key gene signatures. The diagnostic performance was evaluated by ROC curve analysis. An OVA-induced murine asthma model with LPS or poly(I:C) co-administration was established to validate the Expression of the ER stress-related biomarkers.

**Results:**

By integrating bioinformatic analysis of the GEO dataset GSE76262 with machine learning approaches, we identified two ER stress-related key genes, *endoplasmic reticulum to nucleus signaling 1 (ERN1)* and *transmembrane protein 129 (TMEM129)*, whose combined signature can distinguish asthma from healthy controls with an AUC of 0.811. The diagnostic performance of the *ERN1/TMEM129* signature was independently validated in an external cohort (GSE256534), achieving an AUC of 0.907. In human induced sputum, *ERN1* was upregulated while *TMEM129* was downregulated (*P* < 0.001). These changes were validated in an ovalbumin-induced murine asthma model: lung inositol-requiring protein 1 (IRE1, protein encoded by *ERN1*) levels were upregulated while *TMEM129* mRNA was downregulated versus saline controls. Co-exposure to lipopolysaccharides (LPS) or poly(I:C) potentiated airway inflammation and elevated the ER stress chaperone BiP/GRP78; however, only IRE1 levels increased further, whereas *TMEM129* remained unchanged upon LPS and poly(I:C) co-administration.

**Conclusion:**

We identify *ERN1* and *TMEM129* as a two-gene ER stress signature associated with asthma and propose them as potential diagnostic biomarkers for asthma.

## 1 Introduction

Asthma is a heterogeneous inflammatory airway disease whose prevalence continues to rise worldwide (1, 2). Although inhaled corticosteroids and β2-agonists achieve adequate control in most patients, moderate-to-severe asthma remains responsible for the majority of disease-related morbidity, mortality and healthcare costs (3, 4).

Acute exacerbations are defined as acute deteriorations in respiratory symptoms requiring systemic corticosteroids or hospitalization. Acute exacerbations are frequently triggered by allergens, air pollutants, and respiratory viral and bacterial infections. Infections with rhinovirus, influenza, and bacterial lipopolysaccharides (LPS) not only trigger acute attacks but also drive corticosteroid resistance (5–7). Despite advances in understanding how these triggers promote acute asthma exacerbations, the underlying molecular mechanisms remain incompletely understood.

Endoplasmic reticulum (ER) stress occurs when the ER cannot fold proteins properly, and it promotes airway inflammation and asthma (8, 9). Acute exacerbations-often precipitated by allergens, air pollutants or microbial stimuli-are the main drivers of asthma mortality, hospitalization and healthcare costs, and are frequently corticosteroid resistant. When ER homeostasis is disturbed, misfolded proteins accumulate and trigger the unfolded protein response (UPR) (10). Protein kinase R-like ER kinase (PERK), inositol-requiring protein 1 (IRE1), and activating transcription factor 6 (ATF6) are three ER membrane sensors that govern the UPR (8, 11). Examining ER stress can improve our understanding of exacerbation biology and identifies novel diagnostic biomarkers.

Accordingly, this study aimed to identify key ER stress-related genes in asthma and exacerbation using bioinformatics and machine learning, and to develop a diagnostic model for early detection.

## 2 Materials and methods

### 2.1 Dataset collection

Dataset GSE76262 (GPL13158 platform) and GSE256534 were downloaded from the GEO database. The dataset GSE76262 contains induced-sputum gene-expression profiles of 118 moderate-to-severe asthma cases and 21 healthy controls from U-BIOPRED. GSE256534 contains RNA-sequencing profiles of 19 subjects with asthma exacerbation and 13 healthy controls. A list of 267 ER stress-related genes was downloaded from the MSigDB.

### 2.2 Identification of asthma-associated ER stress genes

We employed GEO2R to identify differentially expressed genes (DEGs) between asthma patients and healthy controls (|log_2_FC| > 0.585 and adjusted *P* < 0.05), with Benjamini-Hochberg false discovery rate (FDR) correction for multiple testing. Then the resulting DEGs were intersected with the 267 ER stress-related genes to yield the asthma-relevant ER stress gene set. Functional enrichment analyses of these genes were subsequently performed by Kyoto Encyclopedia of Genes and Genomes (KEGG) pathway and Gene Ontology (GO) enrichment analyses using the GOplot package in R. Gene co-expression network construction was performed using the R package WGCNA.

### 2.3 Protein-protein interaction network construction

Protein-protein interaction (PPI) network construction for candidate ER stress genes was performed via the STRING online database. The top 10 hub genes were then identified by the CytoHubba plugin (MCC algorithm) in Cytoscape 3.10. These 10 genes were subsequently submitted to the GENEMANIA online database for comprehensive interaction and regulatory network predictions.

### 2.4 Machine learning-driven key gene selection and diagnostic validation

Least absolute shrinkage and selection operator (LASSO) logistic regression and random forests (RF) were jointly applied to screen for novel and key asthma signatures. RF and LASSO logistic regression models were constructed using the “randomForest” and “glmnet” packages in R, respectively. For LASSO logistic regression, we used the cv.glmnet function, which performs 10-fold cross-validation by default to select the optimal regularization parameter (λ) and prevent overfitting during model training. For RF, we performed recursive feature elimination with cross-validation using the rfcv function (randomForest package), with 5-fold cross-validation repeated 5 times to ensure robustness in feature selection. Overlapping genes were determined between LASSO logistic regression and RF. Receiver operating characteristic (ROC) curve analysis was performed to validate the diagnostic potential of these hub genes.

### 2.5 Experimental animals

Male BALB/c mice (6-week-old, 18 ± 2 g) were purchased from Beijing HFK Bio-Technology Co., Ltd. (Beijing, China) and housed under specific-pathogen-free conditions (22 ± 2°C, 55 ± 5% humidity, 12-h light/dark cycle) with free access to standard chow and water. Ethical approval for all experimental procedures was obtained from the Institutional Animal Care and Use Committee of Beijing Chao-Yang Hospital (approval No. 25–2095). Four experimental groups were established: Control, ovalbumin (OVA), OVA/LPS, and OVA/Poly(I:C). Model establishment and tissue collection were performed as described previously (5). Briefly, the mice were intraperitoneally sensitized with 100 μg OVA (Sigma-Aldrich, St Louis, MO, United States) mixed with aluminum hydroxide (Thermo Fisher Scientific, Waltham, MA, United States) on days 1 and 7, followed by daily intranasal challenge with 20 μg OVA from days 14 to 16. To mimic infection-triggered exacerbations, separate groups received a single intranasal dose of 50 ng LPS (Sigma-Aldrich, St Louis, MO, United States) or 100 μg poly(I:C) concurrently with each OVA intranasal instillation on days 14–16. Control animals received saline alone.

### 2.6 Histological analysis

Lung sections (4 μm) underwent deparaffinization, rehydration, and hematoxylin-eosin staining. Specifically, sections were treated with xylene, graded ethanol, and distilled water, then stained with hematoxylin (5 min), differentiated in 1% acid alcohol, blued in 0.2% ammonia water, counterstained with eosin (2 min), and finally dehydrated, cleared, and mounted.

### 2.7 Immunohistochemistry

Immunohistochemistry (IHC) was performed on 4-μm-thick formalin-fixed, paraffin-embedded lung sections. After deparaffinization and rehydration, antigen retrieval was carried out by heating slides in 10 mM citrate buffer (pH 6.0) at 95°C for 20 min. Endogenous peroxidase was quenched with 3% H_2_O_2_ in methanol for 10 min at room temperature, followed by three washes with PBS. Non-specific binding was blocked with 5% normal goat serum in PBS for 30 min. Sections were then incubated overnight at 4°C with primary anti-IRE1 (abcam, Cambridge, United Kingdom) and anti-BiP antibodies (Cell Signaling Technology, Beverly, MA, United States) (diluted in PBS containing 1% BSA). After three PBS washes, biotinylated secondary antibody was applied for 1 h at 37°C, followed by streptavidin-HRP complex for 30 min. Immunoreactivity was visualized with 3,3′-diaminobenzidine (DAB) substrate, and nuclei were counterstained with hematoxylin. Slides were dehydrated, cleared, and mounted with neutral balsam for light-microscopy evaluation.

### 2.8 Reverse transcription-quantitative PCR (RT-qPCR)

cDNA was synthesized from mouse lung RNA and bronchoalveolar lavage fluid (BALF) cells using FastKing RT kit (Tiangen, Beijing, China), followed by PCR with SYBR Premix Ex Taq kit (Tiangen, Beijing, China). Primers: murine *GAPDH*: AATGGATTTGGACGCATTGGT (F), TTTGCACTGGTACGTGTTGAT (R); murine *TMEM129*: ACAGTGCAGAATCTGTTGTCG (F), TGCCCATGTAGTAGC CTAACG (R); murine *ERN1*: ACACCGACCACCGTATCTCA (F), CTCAGGATAATGGTAGCCATGTC (R); murine *ATF6*: TCGCCTTTTAGTCCGGTTCTT (F), GGCTCCATAGGTCTG ACTCC (R); murine *XBP1s*: CTGAGTCCGCAGCAGGTG (F), GGCAACAGTGTCAGAGTCCA (R).

### 2.9 Statistical analysis

Bioinformatic analyses were performed in RStudio (v4.4.1). The expression levels of *ERN1* and *TMEM129* in GSE76262 and GSE256534, and RT-qPCR data from mouse experiments were analyzed using GraphPad Prism. Student’s *t*-test compared two groups; one-way ANOVA compared multiple groups followed by Bonferroni correction. A *P-*value of 0.05 or lower was considered statistically significant.

## 3 Results

### 3.1 Identifying ER stress-related DEGs in moderate-to-severe asthma

**FIGURE 1:**
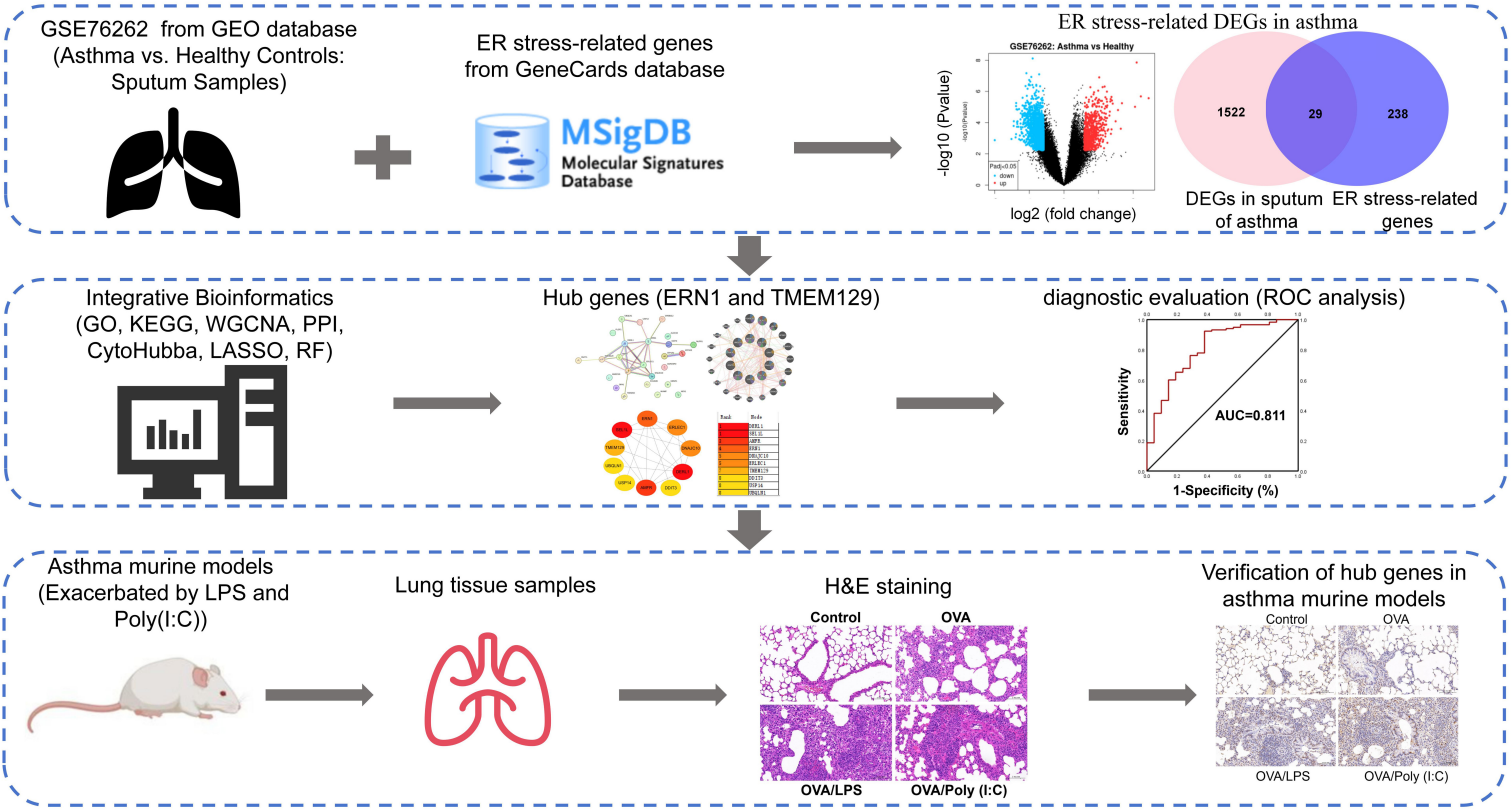
The primary study design of this study.

**FIGURE 2:**
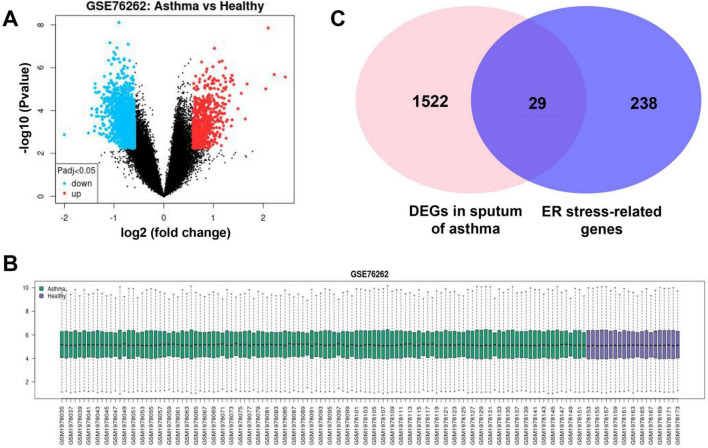
Identification of ER stress-related DEGs in asthma exacerbations. **(A)** Volcano plots of DEGs in GSE76262. **(B)** Box plots of the normalized data in GSE76262. **(C)** Venn diagram showing the overlap between DEGs identified from the GSE76262 dataset and the 267 ER stress-related genes obtained from the MSigDB database.

The primary study design is in Figure 1. To uncover genes linked to ER stress in asthma, we analyzed the sputum-transcriptome dataset GSE76262 from the GEO database (118 moderate-to-severe asthma and 21 healthy controls). DEGs were identified with GEO2R (adj. *P* < 0.05 and |log_2_FC| > 0.58) (Figure 2A). Box plots confirmed high degree of consistency across samples (Figure 2B). A list of 267 ER stress-related genes was obtained from MSigDB. Overlap analysis showed 29 ER stress-related DEGs (Figure 2C).

### 3.2 Functional enrichment analysis of ER stress-related genes in moderate-to-severe asthma

**FIGURE 3:**
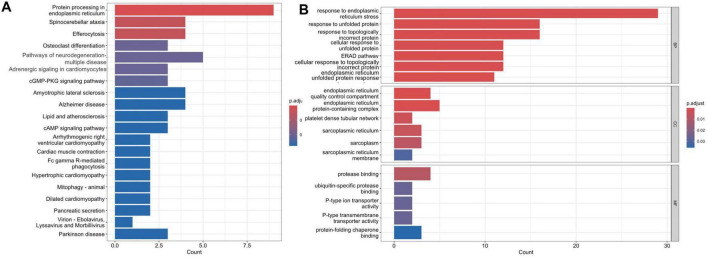
Functional enrichment analysis of the ER stress-related DEGs from GSE76262. **(A)** KEGG analysis of the ER stress-related DEGs. **(B)** GO analysis of the ER stress-associated DEGs.

To explore the biological roles of the 29 ER stress-related DEGs, we performed KEGG and GO enrichment analyses. KEGG pathways were most enriched in protein processing in ER, efferocytosis, osteoclast differentiation, and neurodegeneration-related cascades (Figure 3A). GO analysis revealed that the main enriched BP terms included ER stress, response to unfolded protein, and ER-associated degradation (ERAD) pathway (Figure 3B). The primary CC terms included the ER quality-control compartment and sarcoplasmic reticulum (Figure 3B), while MF terms were dominated by protease binding, ubiquitin-specific protease binding, and protein-folding chaperone binding (Figure 3B).

### 3.3 Identification of co-expression gene modules

**FIGURE 4:**
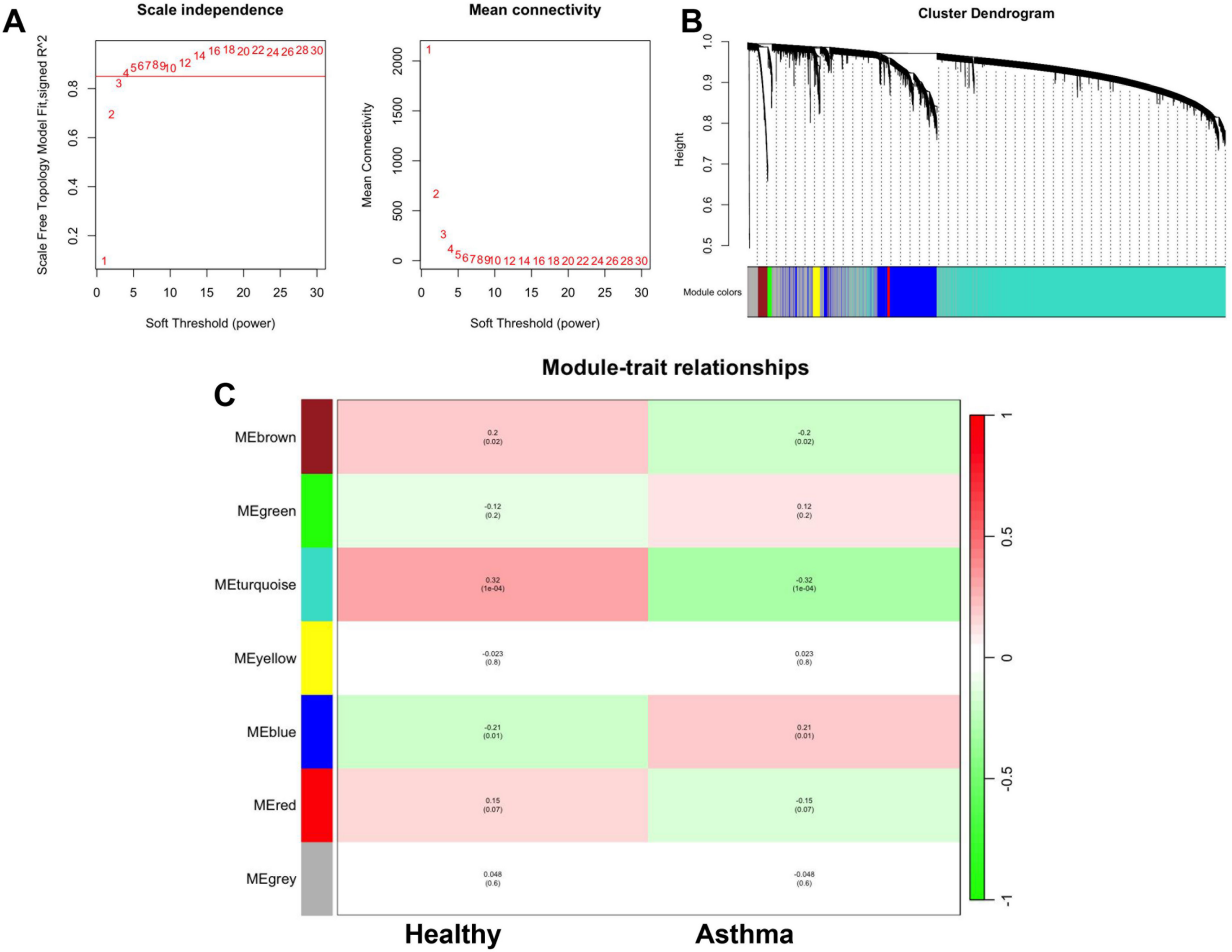
WGCNA analysis of GSE76262. **(A)** The selection of the optimal soft threshold. **(B)** The dendrogram of clustering and its associated module colors. **(C)** The correlation between module eigengenes and clinical features (asthma or healthy).

We performed WGCNA on GSE76262 to screen for modules linked to asthma. Asoft-threshold power of β = 4 (scale independence of *R*^2^ > 0.8) was chosen (Figure 4A). A total of seven co-expression modules were obtained (Figure 4B). Module-trait correlation analysis revealed that the blue, brown and turquoise modules were significantly associated with asthma (*P* < 0.05; Figure 4C). We next intersected the WGCNA-significant modules with the 29 ER stress-associated DEGs, yielding 25 candidate genes.

**FIGURE 5:**
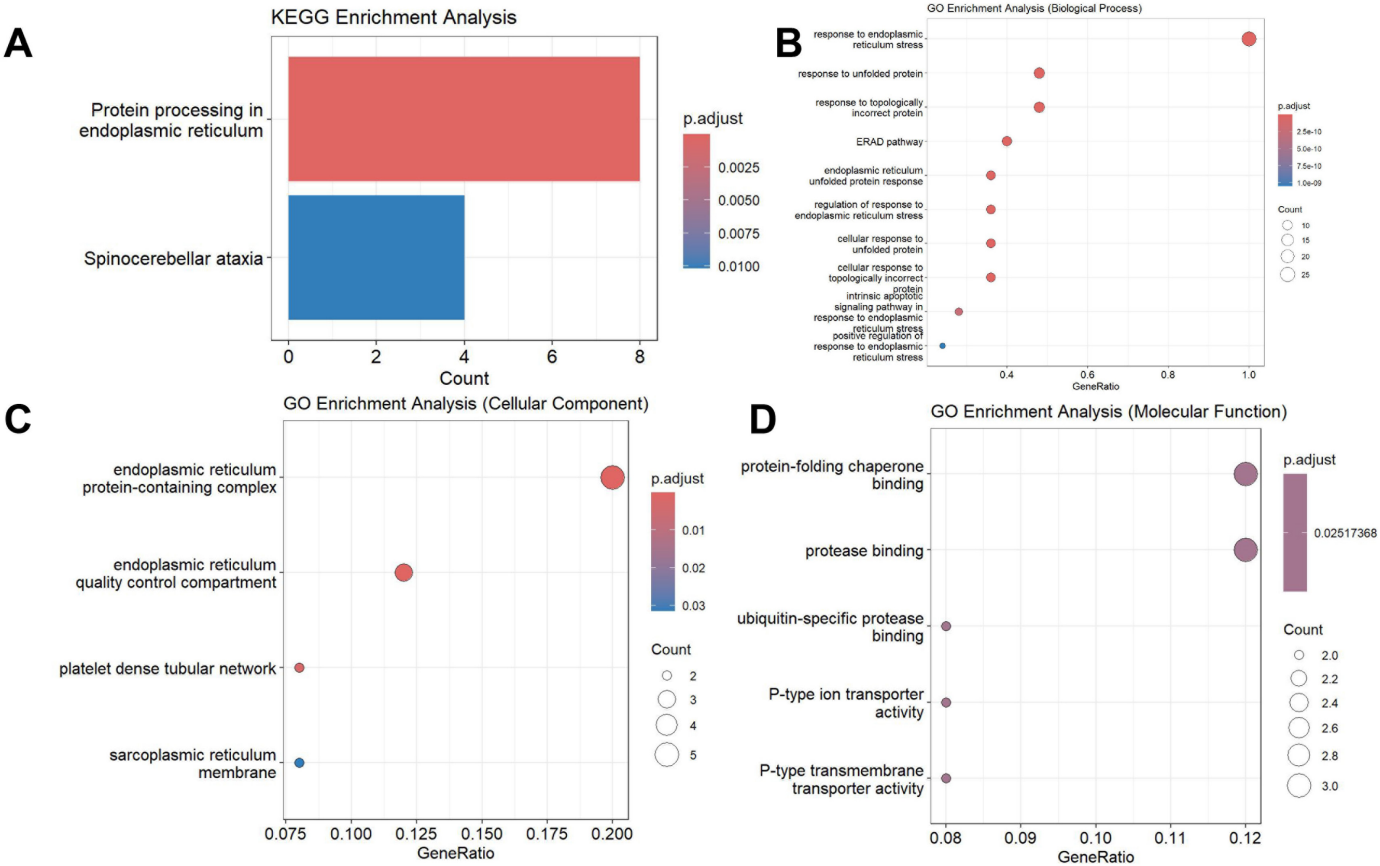
Functional enrichment analysis of the 25 candidate genes. **(A)** KEGG analysis of the 25 candidate genes. **(B–D)** GO analysis of the 25 candidate genes: BP **(B)**, CC **(C)**, and MF **(D)**.

Functional enrichment of the 25 candidate genes revealed that KEGG pathways were most enriched in protein processing in ER and spinocerebellar ataxia (Figure 5A). GO analysis revealed that the main enriched BP terms included response to ER stress, response to unfolded protein, response to topologically incorrect protein, ERAD pathway, and ER unfolded protein response (Figure 5B); The primary CC terms included ER protein-containing complex, ER quality control compartment, platelet dense tubular network, and sarcoplasmic reticulum membrane (Figure 5C); MF terms were dominated by protein-folding chaperone binding, protease binding, ubiquitin-specific protease binding, P-type ion transporter activity, and P-type transmembrane transporter activity (Figure 5D).

### 3.4 Constructing the ER stress-related hub gene network in asthma

**FIGURE 6:**
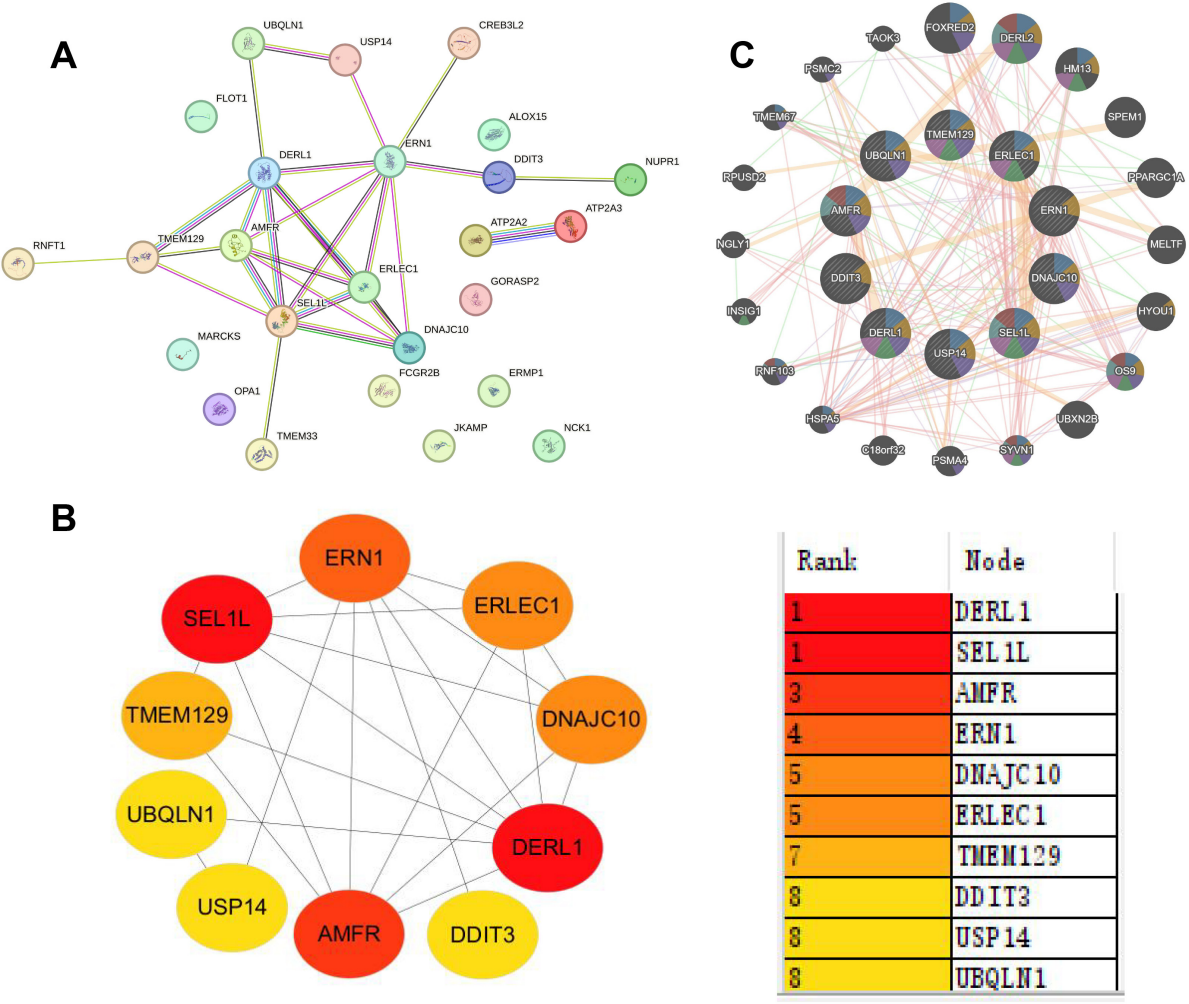
Network analysis of the 25 candidate genes. **(A)** PPI network of 25 candidate genes built with STRING. Nodes represent proteins. PPI enrichment *P* < 1.0 × 10^–16^. **(B)** Top 10 hub genes ranked by MCC using CytoHubba in Cytoscape. **(C)** Interacting proteins predicted by the GENEMANIA database for the key genes identified in the PPI network.

We first constructed a PPI network of the 25 ER stress-related genes using STRING (Figure 6A; 27 edges, 25 nodes, *P* < 1.0 × 10^–16^). The top-10 hub genes identified by MCC (CytoHubba) were *DERL1, SEL1L, AMFR, ERN1, DNAJC10, ERLEC1, TMEM129, DDIT3, USP14*, and *UBQLN1* (Figure 6B). To explore broader regulatory context, we expanded these hub genes in GeneMANIA, which yielded a 30-gene network (10 original genes + 20 predicted) with 220 edges (co-expression, pathway and physical interaction; Figure 6C).

### 3.5 Screening and validation of critical gene signatures

**FIGURE 7:**
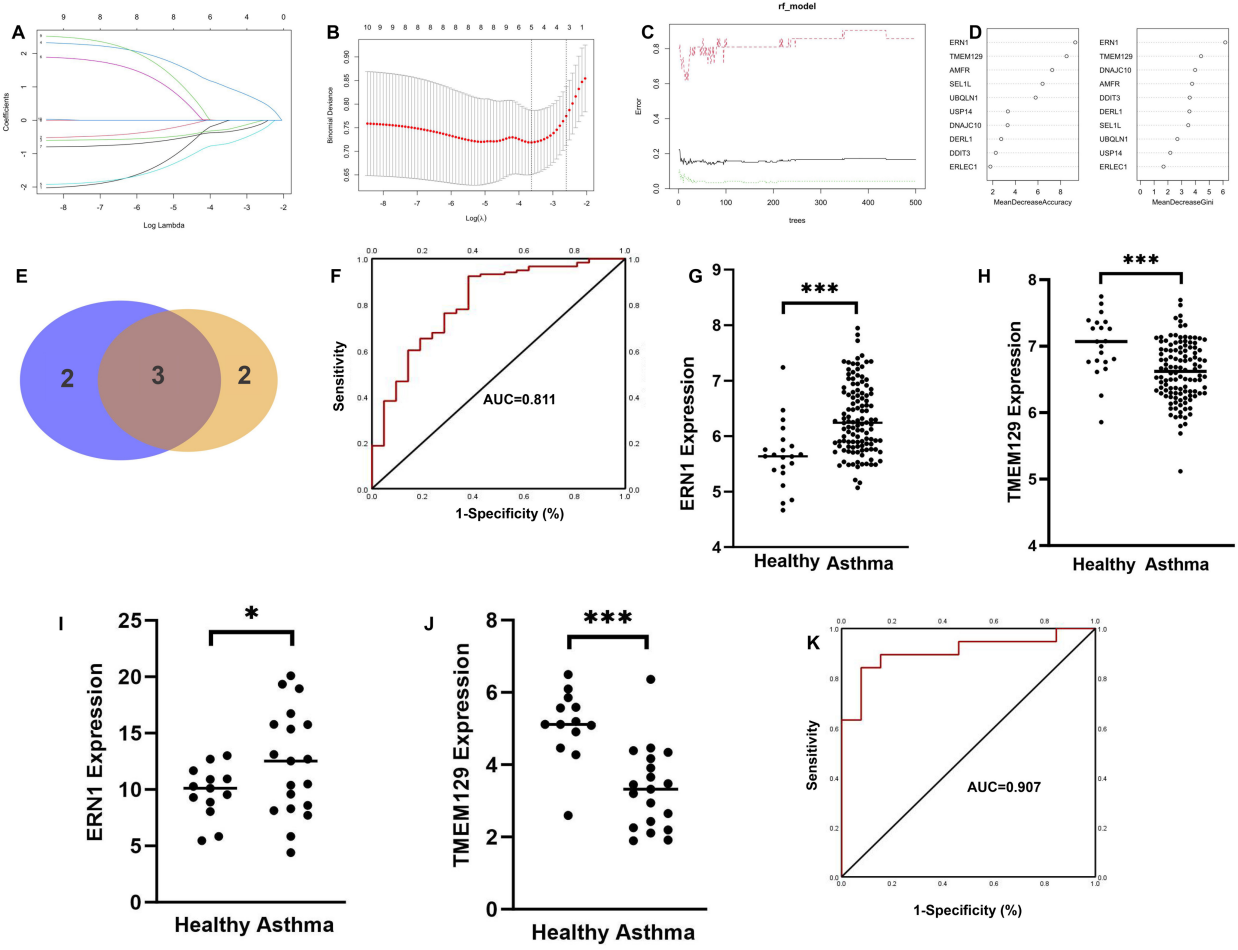
Screening of critical signatures *via* multiple machine-learning algorithms. **(A,B)** LASSO regression analysis: **(A)** coefficient profiles of the 10 potential hub genes. **(B)** Tuning parameter (lambda) selection in the LASSO model. **(C)** RF trees. **(D)** Variable importance, as measured by the mean decrease in accuracy (left panel) or the Gini coefficient (right panel). **(E)** Venn diagram shows the intersection of critical signatures obtained by RF and LASSO regression analysis. **(F)** AUC of the 2 hub genes in GSE76262. **(G)** The expressions of *ERN1* in GSE76262. **(H)** The expressions of *TMEM129* in GSE76262. **(I)** The expression validation of *ERN1* in GSE256534. **(J)** The expression validation of *TMEM129* in GSE256534. **(K)** ROC curves for the 2 hub genes in validation set GSE256534. *P ≤ 0.05, ***P ≤ 0.001.

The ten hub genes were next subjected to LASSO logistic regression and RF feature selection. Five genes were determined by LASSO logistic regression algorithm (*DERL1, AMFR, ERN1, DNAJC10* and *TMEM129*) (Figures 7A,B). The RF algorithm scored the 10 genes for importance and identified the five highest-importance ones (*ERN1, TMEM129, AMRF, SEL1L*, and *UBQLN1*) (Figures 7C,D). The intersection of these two sets yielded the ER stress-related signatures for asthma (*ERN1, TMEM129*, and *AMRF*) (Figure 7E). The AUC for the three-gene set was 0.817, showing only a marginal improvement compared with 0.811 for the two-gene combination (*ERN1* and *TMEM129*) (Figure 7F). Among the five LASSO-selected genes, *ERN1* exhibited the strongest effect (β = 1.03), while *TMEM129* (β = −0.35) and *AMFR* (β = −0.28) showed modest protective effects. Furthermore, *AMFR* ranked third in RF feature importance, below *ERN1* and *TMEM129*, and its inclusion provided only a minimal incremental AUC gain (0.006). Notably, *AMFR* upregulation in asthma has been reported specifically in mouse lung macrophages (12), whereas our dataset reflects mixed-cell populations. The same study showed no significant *AMFR* changes in eosinophils, neutrophils, or dendritic cells, suggesting strong cell type-specific regulation that may limit its generalizability across different tissue compositions and complicate experimental validation in non-macrophage-dominant models. Given its (1) suboptimal RF ranking, (2) negligible incremental AUC contribution, (3) cell type-specific expression pattern limiting broad applicability, and (4) the principle of model parsimony, we retained the two highest-importance RF-ranked genes (*ERN1* and *TMEM129*). We verified the expression of *ERN1* and *TMEM129* in GSE76262. *ERN1* was significantly up-regulated (Figure 7G), whereas *TMEM129* was significantly down-regulated (Figure 7H), in induced sputum from moderate-to-severe asthma patients compared with healthy controls.

We next employed GSE256534 as an external validation dataset (19 asthma exacerbation and 13 healthy controls) to verify the expression patterns of *ERN1* and *TMEM129*. Consistent with the GSE76262 cohort, *ERN1* was significantly up-regulated, whereas *TMEM129* was significantly down-regulated, in asthma patients compared with healthy controls (Figures 7I,J). The two-gene combination achieved an AUC of 0.907 in distinguishing asthma from healthy subjects in this independent cohort (Figure 7K), confirming the diagnostic utility of the *ERN1-TMEM129* signature.

### 3.6 Establishment of OVA-induced asthma models and viral/bacterial exacerbation

**FIGURE 8:**
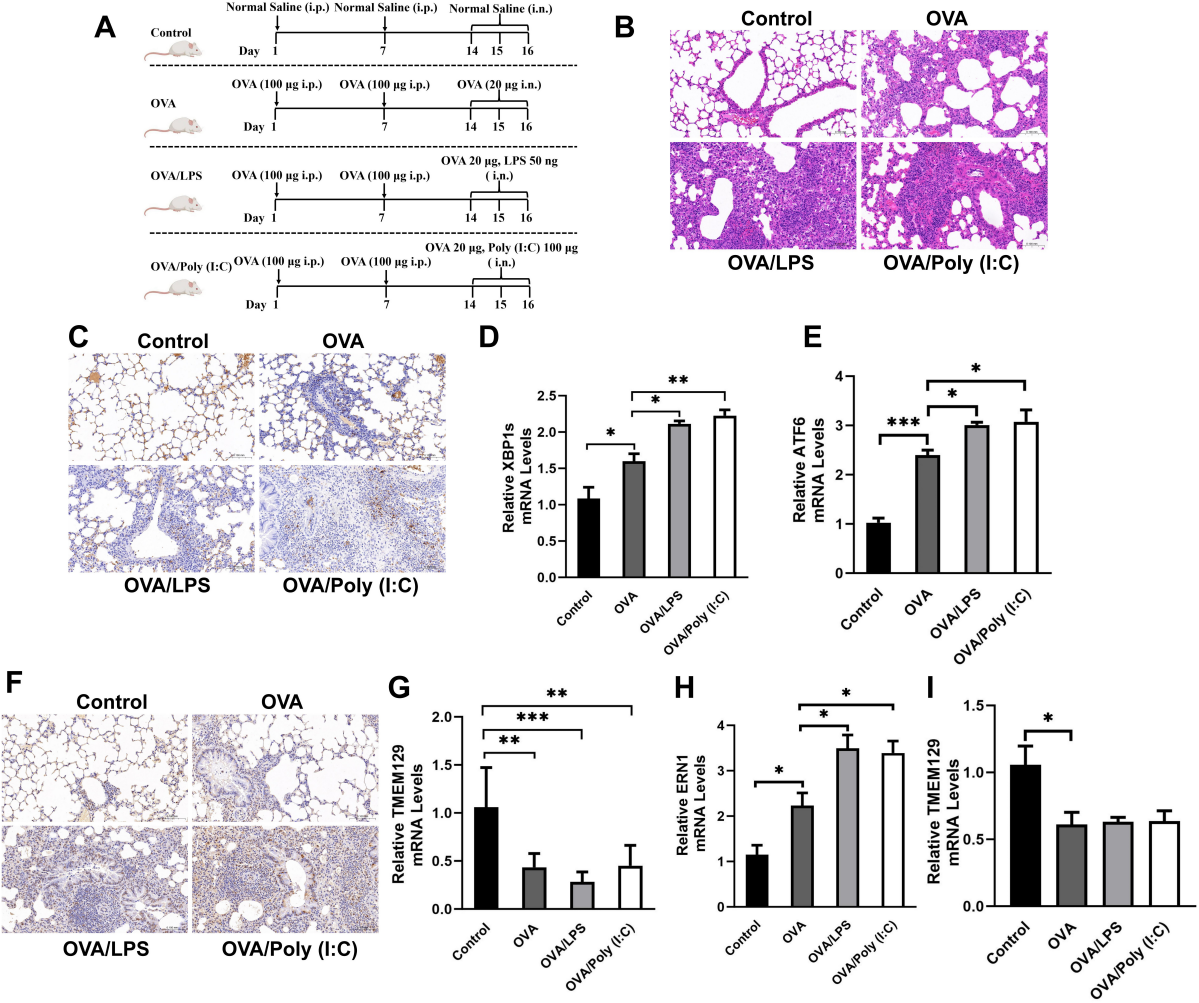
Expression of ER stress-related protein, and *ERN1* and *TMEM129* in asthma murine models. **(A)** Experimental design. **(B)** H&E staining. **(C)** BiP in lung tissues detected by immunohistochemical staining. **(D)** *XBP1s* in lung tissues detected by RT-qPCR. **(E)** *ATF6* in lung tissues detected by RT-qPCR. **(F)** IRE1 in lung tissues detected by immunohistochemical staining. **(G)** *TMEM129* in lung tissues detected by RT-qPCR. **(H)** *ERN1* in BALF cells detected by RT-qPCR. **(I)** *TMEM129* in BALF cells detected by RT-qPCR. *P ≤ 0.05, **P ≤ 0.01, ***P ≤ 0.001.

To functionally validate the ER stress-related signatures identified in human sputum, we established an OVA induced asthma murine model as described in methods (Figure 8A). Immunohistochemistry for the ER-luminal chaperone BiP (GRP78) was used to measure ER stress levels in lung sections. H&E staining showed marked peribronchial and perivascular inflammation in OVA-treated mic (Figure 8B). Moreover, additional exposure to LPS or poly(I:C) further amplified inflammatory infiltrates (Figure 8B). Immunohistochemical staining demonstrated marked up-regulation of BiP/GRP78 in lung tissues of OVA-treated mice relative to controls (Figure 8C). LPS or poly(I:C) co-administration further elevated BiP expression compared with the OVA group (Figure 8C). *XBP1s* and *ATF6* mRNA levels in whole-lung homogenates were measured by RT-qPCR. Consistent with BiP upregulation, *XBP1s* was significantly induced in OVA-challenged mice relative to saline controls (Figure 8D), and further elevated by LPS or poly(I:C) co-exposure (Figure 8D). Similarly, *ATF6* expression showed a significant increase in the OVA group (Figure 8E), with additional augmentation following LPS or poly(I:C) administration (Figure 8E). Collectively, these data show that OVA-challenged asthmatic mice display elevated pulmonary ER stress, which is further amplified by LPS or poly(I:C) co-exposure.

IRE1 is encoded by the *ERN*1 gene. IRE1 and *TMEM129* expression was quantified in lung tissues from all four experimental groups to validate key ER stress genes in our asthma models. Immunohistochemistry results revealed that, compared with saline-treated controls, OVA-sensitized/challenged mice exhibited a significant increase in pulmonary IRE1 levels (Figure 8F). Co-administration of LPS or poly(I:C) further elevated IRE1 expression (Figure 8F). Because a validated commercial antibody for murine TMEM129 is unavailable, we quantified its transcript levels by RT-qPCR in whole-lung homogenates. *TMEM129* mRNA was significantly reduced in OVA-challenged mice compared with saline controls (Figure 8G). However, LPS and poly(I:C) co-administration showed no effect on *TMEM129* expression (Figure 8G).

Moreover, the mRNA levels of *ERN1* and *TMEM129* in BALF cells from our mouse models were measured by RT-qPCR. In BALF cells, *ERN1* mRNA was significantly upregulated in OVA-challenged mice relative to saline controls (Figure 8H), with further elevation upon LPS or poly(I:C) co-administration (Figure 8H). Similarly, *TMEM129* mRNA was significantly reduced in OVA-challenged BALF cells (Figure 8I), and LPS or poly(I:C) co-exposure showed no additional effect (Figure 8I), recapitulating the expression pattern observed in whole-lung homogenates.

## 4 Discussion

Our data demonstrated that ER stress was intrinsically heightened in asthma and was further amplified by LPS or poly(I:C) exposure. Comprehensive bioinformatics analyses identified *ERN1* and *TMEM129* as central regulators of this exacerbation-driven ER stress response.

ER is the main factory for folding secretory and trans-membrane proteins (13). When misfolded or unfolded proteins accumulate beyond the ER’s folding capacity, ER stress is triggered, disrupting ER homeostasis and contributing to diabetes, neurodegeneration, asthma, COPD and other disorders (14–17). ER stress is now recognized as a key driver of airway inflammation, lung injury, mucus hyper-secretion and airway remodeling (8, 18, 19). The ER-resident chaperone BiP (GRP78) is a master regulator of ER functions (20). Its dysregulation is defined as an important marker of ER stress in several studies (21–24). In our OVA-induced asthma model, pulmonary BiP levels were markedly higher than in saline controls, confirming elevated ER stress in allergic airways. Microbial exposures-such as respiratory viruses and bacterial products-have been increasingly recognized as key drivers of both the initiation and acute exacerbation of asthma. We co-exposed asthmatic mice to LPS or poly(I:C), both stimuli further amplified inflammation and raised BiP levels, indicating aggravated ER stress. To identify potential ER stress-related biomarkers in asthma, we conducted bioinformatics analysis of GEO datasets and subsequently validated the candidate key genes using *in vivo* murine asthma models.

Using GSE76262 and the ER stress gene set from MSigDB, we identified 47 ER stress-related genes dysregulated in asthma. Subsequent WGCNA, PPI/CytoHubba hub-gene analysis and machine-learning refinement identified *ERN1* and *TMEM129* as potential biomarkers. ROC analysis showed that this two-gene signature significantly discriminates asthmatic patients from healthy controls, highlighting *ERN1* and *TMEM129* as promising diagnostic biomarkers for asthma. *ERN1* encodes the UPR sensor IRE1. We next validated the expression changes of *ERN1* and *TMEM129* in the OVA-induced asthma murine model. Consistent with the human data, the protein levels of IRE1 were markedly up-regulated, whereas *TMEM129* was transcriptionally repressed. To mimic bacterial or viral infection-major triggers of acute exacerbations, mice were co-exposed to LPS or poly(I:C). Both stimuli further elevated IRE1 levels, yet failed to reduce *TMEM129* expression further.

*ERN1* encodes the UPR sensor IRE1 and serves as a vital transmembrane ER stress sensor. Over the past decade or two, the IRE1 pathway has emerged as a significant player in various diseases, including asthma by regulating inflammation levels, immunity, and lipid metabolism (25, 26). Consistent with our human data, OVA-challenged mice showed marked IRE1 induction that was further enhanced by LPS or poly(I:C).

*TMEM129* encodes ER-anchored E3 ubiquitin ligase TM129, which mediates protein degradation via regulated proteolysis and the UPR (27, 28). In the present study, *TMEM129* expression was repressed in both human sputum and murine asthma lung tissues. It’s down-regulation may impair clearance of misfolded proteins (28), thereby sustaining chronic ER stress. Notably, although LPS or poly(I:C) co-exposure markedly worsened airway inflammation, it did not lower *TMEM129* expression further. Liu X et al. reported that *TMEM129* acts as the virus-recruited E3 ligase that ubiquitinates FcRn within the ER, leading to its dislocation and degradation (29). Virus-induced degradation of MHC-I also depends on *TMEM129* (30). However, none of these studies examined whether viral infection alters overall *TMEM129* expression. Thus, changes in its cellular level during infection remain unknown.

Despite its important role in ER-associated protein degradation, *TMEM129* remains largely unexplored in human disease. To date, few reports have linked this E3 ligase to pathological conditions. In osteoarthritis, genome-wide analyses have positioned *TMEM129* as an effector gene of genetic risk: Brumwell et al. demonstrated that individuals carrying methylation marks that reduce *TMEM129* expression exhibit a significantly higher incidence of osteoarthritis (27). Protein degradation within the ER is essential for cartilage homeostasis, and its imbalance is increasingly recognized as a driver of osteoarthritis. Brumwell et al. therefore hypothesized that reduced *TMEM129* expression undermines the UPR, which allows misfolded proteins to accumulate and thereby increases osteoarthritis susceptibility. GWAS identified the *TMEM129* intronic variant rs56337234 as a shared susceptibility locus for both type 1 and type 2 diabetes (31). Additionally, *TMEM129* has been linked to bladder (32, 33), ovarian (34) and lung adenocarcinoma (35) by methylation arrays, bioinformatics analyses and GWAS, yet the mechanisms underlying these associations remain unexplored. To our knowledge, *TMEM129* has not previously been examined in asthma, COPD or other airway inflammatory diseases. We now show that its abundance is markedly reduced in induced sputum from patients with moderate-to-severe asthma and in lung tissue from OVA-sensitized/challenged mice, mirroring the down-regulation observed in osteoarthritis. As in earlier reports, however, our findings are descriptive; functional studies are required to determine how *TMEM129* down-regulation sustains ER stress and amplifies allergic airway inflammation.

This study has several limitations. Firstly, although key genes were identified by analyzing publicly available GEO dataset and validated in murine asthma models, their clinical utility as biomarkers for human asthma remains untested. Prospective studies should collect sputum and blood samples to determine whether *ERN1* and/or *TMEM129* levels correlate with disease severity, corticosteroid responsiveness, or exacerbation risk. Secondly, this study aimed to develop a predictive gene signature for asthma diagnosis. While our findings demonstrate a robust association between the *ERN1/TMEM129* signature and asthma in both human and murine samples, they do not establish a causal role for either gene in disease initiation or progression. Functional studies using conditional knockout or transgenic over-expression mouse lines are required to clarify the roles of *ERN1* and *TMEM129* in airway inflammation in future investigations.

## Data Availability

The datasets presented in this study can be found in online repositories. The names of the repository/repositories and accession number(s) can be found at: https://www.ncbi.nlm.nih.gov/geo/, GSE76262.
